# methCancer-gen: a DNA methylome dataset generator for user-specified cancer type based on conditional variational autoencoder

**DOI:** 10.1186/s12859-020-3516-8

**Published:** 2020-05-11

**Authors:** Joungmin Choi, Heejoon Chae

**Affiliations:** Division of Computer Science, Sookmyung Women′s University, Seoul, Republic of Korea

**Keywords:** DNA methylation, Cancer, Generator, Conditional variational autoencoder, Simulator

## Abstract

**Background:**

Recently, DNA methylation has drawn great attention due to its strong correlation with abnormal gene activities and informative representation of the cancer status. As a number of studies focus on DNA methylation signatures in cancer, demand for utilizing publicly available methylome dataset has been increased. To satisfy this, large-scale projects were launched to discover biological insights into cancer, providing a collection of the dataset. However, public cancer data, especially for certain cancer types, is still limited to be used in research. Several simulation tools for producing epigenetic dataset have been introduced in order to alleviate the issue, still, to date, generation for user-specified cancer type dataset has not been proposed.

**Results:**

In this paper, we present methCancer-gen, a tool for generating DNA methylome dataset considering type for cancer. Employing conditional variational autoencoder, a neural network-based generative model, it estimates the conditional distribution with latent variables and data, and generates samples for specified cancer type.

**Conclusions:**

To evaluate the simulation performance of methCancer-gen for the user-specified cancer type, our proposed model was compared to a benchmark method and it could successfully reproduce cancer type-wise data with high accuracy helping to alleviate the lack of condition-specific data issue. methCancer-gen is publicly available at https://github.com/cbi-bioinfo/methCancer-gen.

## Background

DNA methylation is one of the epigenetic mechanisms, playing a critical role in various biological processes, such as gene regulation, cell differentiation, and suppression of transposable elements [1–3]. Recent studies have reported that diverse types of neoplasia and cancer are related to changes in DNA methylation [4] and abnormal DNA methyl patterns are considered one of the biomarkers for diagnosing cancer [5, 6]. In addition, the tissue-specific DNA methylation patterns determine the origin of the cancer [7].

To satisfy growing needs for better diagnosis and advance understanding of driver mutations leading to uncontrolled cell growth and tumor formation, increasing amounts of genomic and epigenomic data have been publicly available through large-scale projects aimed for comprehensive integrated analysis of cancer [8]. The Cancer Genome Atlas (TCGA) program provided a collection of multi-platform molecular profiles across 33 different cancer types, composed of various clinical and genomic datasets [9]. Based on the multi-omics integrated analysis, evidence for biological mechanism in cancers was provided. ENCyclopedia of DNA elements (ENCODE) project [10] and Roadmap Epigenomics Mapping Consortium [11] produced public human epigenetic resources to investigate cancer biology. Through these projects, the identification of functional elements in the human genome sequence has been made. Utilizing public cancer resources, studies have focused on discovering the relationship between DNA methylation signature and cancer. MethyCancer presented and analyzed an integrated dataset of DNA methylation, mutation and gene expression profiling for tumor cells with cancer information [12]. MethHC provided a systematic integration comprising DNA methylation and mRNA/microRNA profiles in normal and tumor tissues and demonstrated epigenetic patterns for cancer prognosis [13]. MethCNA introduced a comprehensive database of DNA methylation and copy number alterations, which assisted to explore epigenetic patterns and identify key factors in cancer [14]. However, most public methylome dataset utilized in research, are still limited to the above major repositories.

To overcome the limitation of public data, computational approaches for generating methylome dataset have been introduced to provide methylation levels and reproduce a wide range of experimental setups. M.R.Lacey et al. developed an algorithm for producing methylation profiles based on reduced representation bisulfite sequencing (RRBS) to identify interactions between technical and biological variables among the RRBS dataset analysis [15]. Based on the observation from a subset of samples collected from ENCODE database, parametric models were fit to the distributions of CpG site positions and methylation levels to perform the simulation. DNemulator simulated cytosine methylation rate, sequencing errors and bisulfite conversion by random assignment and change with probability for various bisulfite sequencing experiments based on DNA reads of human reference genome [16]. WGBSSuite was proposed as a simulation tool for single-base DNA methylation data based on whole genome bisulfite sequencing (WGBS), employing two hidden markov models each for CpG location and methylation status [17]. Various experiment setups were reproduced to provide real case scenarios. pWGBSSimla generated WGBS data for a given user-specified genomic region and cell type by simulating methylated read count for specific CpG based on binomial distribution with approximated parameters for read depth and methylation rate of CpG [18]. Although, these simulation tools allow performance comparison among different methylation analysis methods and help to reproduce a wide range of experimental design to support further analysis, however, either they do not provide condition-specific data generation such as cancer type or only allow limited number of pre-defined condition.

In recent years, deep neural network (DNN) based generative model has been presented and achieved remarkable results due to its ability for capturing nonlinear distributed representations [19]. Variational autoencoder (VAE) [20], one of the deep generative model based on variational inference, has been widely adopted for learning latent representations and performing generation task based on trained features [21]. Employing VAE, several studies have been introduced to explore biological features in cancer based on DNA methylation dataset. By learning lower dimensional latent space on methylome data of lung cancers, signals representing each subtype for the sample were profiled [22]. Based on cancer relevant biological features extracted from VAE, breast cancer subtypes were classified to show the effectiveness of unsupervised learning using DNA methylation [23]. A.J. Titus et al. extracted latent features using VAE to investigate a set of CpGs correlated to Estrogen Receptor status [24]. Utilizing DNA methylation dataset, VAE has been employed to identify informative latent variables in the specific type of cancer, however, to the best of our knowledge, simulation of epigenetic dataset conditioned to the designated cancer type based on the generative model has not been presented yet.

In this paper, we propose a methCancer-gen, a tool for generating DNA methylome dataset based on a user-specified cancer type. We employed a conditional variational autoencoder (CVAE) [25], an extension of a standard VAE, suitable for incorporating a control for the condition. It allows generating samples similar but not identical to input data from modeling conditional distribution with latent variables and data. Different from VAE, CVAE has control on the data generation process, therefore by changing the conditional variable which refers to cancer type in our model, DNA methylation simulation data for specified cancer type will be generated. To demonstrate the data simulation of methCancer-gen for the user-specified cancer type, we compared dataset generated from our model to a benchmark method and validated its functionality.

## Results

### Experimental design

#### Benchmark method

To evaluate the methCancer-gen for DNA methylation data generation, a benchmark method for cancer data generation was designed under the assumption that beta values for each CpG site follow a beta distribution [26]. The distributional parameters (*α* and *β*) for each CpG and cancer type were estimated and methylation dataset was simulated from the approximated distribution models. For each cancer type, 100 DNA methylation datasets were generated from methCancer-gen and benchmark method. We compared the accuracies of dataset generated from each method using the most widely used, five different machine learning (ML) based classification algorithms: decision tree (DT) [27], Naive Bayes (NB) [28], random forest (RF) [29], K-nearest neighbor (KNN) [30], and support vector machine (SVM) [31]. This evaluation shows validation of whether the generated cancer dataset is predicted to the intended cancer type we specified to methCancer-gen. Overview of the performance evaluation design is described in Table 1.

**Table 1:** Description of trained models and dataset for simulation evaluation

**TCGA Dataset**	**Training model**	**Description**
70%	methCancer-gen	CVAE based DNN model
	Benchmark	Estimating beta distribution of beta values for each CpG
30%	5 different ML based classifiers	Classifying dataset with 100 generated data for each cancer

#### Dataset

We used a DNA methylome dataset composed of 8,051 primary solid tumor tissue samples from 25 cancer types measured by Illumina Human Infinium 450K assay [32], obtained from TCGA. 70% of dataset was randomly selected and used for generating simulation dataset by training methCancer-gen to learn latent representations and benchmark to estimate the distribution, while 30% was used for training multi-class classifiers for predicting 25 cancer types. Cancer types and the number of samples used for training methCancer-gen and 5 classifiers are listed in Table 2.

**Table 2:** 25 cancer types and the number of samples used for training generators and classifiers

**Cancer type**	**Number of samples for training**	
	**Generators**	**Classifiers**
BLCA	292	126
BRCA	555	238
CESC	214	93
COAD	219	94
ESCA	129	56
GBM	98	42
HNSC	369	159
KIRC	226	98
KIRP	192	83
LGG	361	155
LIHC	263	114
LUAD	331	142
LUSC	259	111
MESO	60	27
PAAD	128	56
PCPG	125	54
PRAD	351	151
READ	68	30
SARC	182	79
SKCM	72	32
STAD	276	119
TGCT	105	45
THCA	354	153
THYM	86	38
UCEC	306	132

### Performance evaluation for the simulation performance

**Fig. 1:**
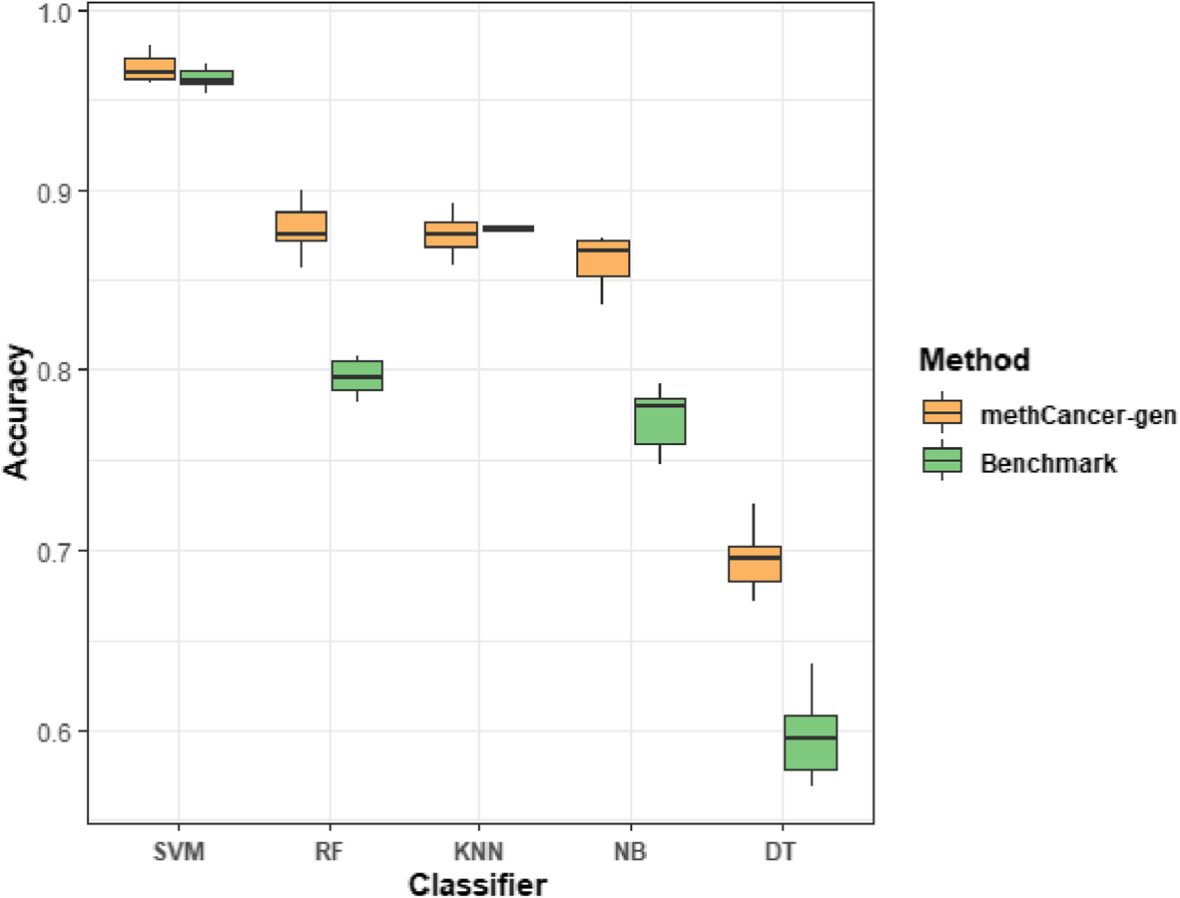
Evaluation of methCancer-gen against benchmark method for accurate data generation. Each boxplot contains accuracies for all the cancer types for each experiment. Accuracy was measured by 5 ML algorithms classifying 25 cancer types of generated dataset

To evaluate the performance of DNA methylation dataset generation of the methCancer-gen for designated cancer type and test the accuracy of simulation data with respect to real data, it was compared to the benchmark method based on estimating beta distribution for each CpG site in each cancer. Based on the preprocessed dataset, both methods generated 100 simulated datasets composed of 394,355 CpGs for each cancer type. Five different multi-class classification algorithms were used to predict cancer types of the simulated dataset from each generation method. The performance was evaluated by measuring average classification accuracy repeated ten times. The evaluation results showed that methCancer-gen outperformed the benchmark, achieving an average classification accuracy of 0.967, 0.875, 0.877, 0.858, and 0.694 for SVM, RF, KNN, NB, and DT, respectively, while benchmark was 0.964, 0.796, 0.875, 0.772 and 0.595, respectively (Fig. 1). The cancer-type wise accuracy and the area under curve (AUC) results are shown in the Supplementary material S1 (Table A) and S2.

Furthermore, we investigated whether a classifier trained using methCancer-gen would improve the classification accuracy compared to a classifier trained with data from TCGA only. For the experiments, three SVM classifiers were trained, where the first model was based on only utilizing 30% of TCGA data and the other two classifiers were trained based on a combined dataset with the same 30% TCGA data and the generated dataset from methCancer-gen and benchmark, respectively. During the experiment, the amount of generated data was gradually increased from 100 to 500 samples for each cancer type (Table 3). 70% of TCGA data used for training methCancer-gen was not included in training SVM classifiers. To evaluate the performance of each SVM classifier, we obtained 1,038 methylation samples of 8 cancer types from methCNA [14], a comprehensive database containing Infinium HumanMethylation450K data resources of human cancer collected from Gene Expression Omnibus database. Each experiment was repeated five times.

**Table 3:** Description of trained models and dataset for usability evaluation

**Classifier**	**Training dataset**	**Testing dataset**
**TCGA only**	30% of TCGA (854 samples)	8 types of cancer dataset from methCNA (1,038 samples)
**TCGA & benchmark**	30% of TCGA & 100-500 generated dataset for each cancer type	
**TCGA & methCancer-gen**		

From the results (Table 4), the classifier utilizing dataset composed of TCGA and 300 generated datasets from methCancer-gen exhibited the highest average accuracy of 0.823 and AUC of 0.914, compared to 0.762 and 0.869 of the benchmark, and 0.751 and 0.864 of TCGA only. The cancer-type wise AUC results are shown in the Supplementary material S3. Moreover, utilizing 300 generated samples for training the SVM classifier achieved a higher average accuracy of 0.823, compared to 0.809 and 0.799 for using 200 and 100 simulation samples, respectively. Increasing the number of generated samples more than 300 for each cancer type did not help to improve the performance of the classifier. Overall, utilizing generated data by methCancer-gen improved the performance of the classifier on 6 of 8 cancer types.

**Table 4:** Comparison of cancer type prediction accuracy for SVM classifiers trained based on different dataset

**Cancer**	Number of testing samples	**TCGA only**	**TCGA & benchmark**			**TCGA & methCancer-gen**		
			Number of generated dataset for each cancer type					
			**100**	**200**	**300**	**100**	**200**	**300**
**BRCA**	313	0.796	0.796	0.796	0.796	0.799	0.802	**0.809**
**COAD**	102	0.922	0.922	0.922	0.922	0.931	0.951	**0.951**
**GBM**	71	**0.972**	0.972	0.972	**0.972**	0.972	0.972	**0.972**
**KIRC**	45	**0.733**	0.733	0.733	**0.733**	0.733	0.733	**0.733**
**LUAD**	162	0.969	1.000	1.000	**1.000**	1.000	1.000	**1.000**
**PAAD**	166	0.139	0.168	0.168	0.175	0.398	0.434	**0.434**
**PRAD**	20	0.700	0.700	0.700	0.700	0.700	0.700	**1.000**
**SKCM**	159	0.868	0.887	0.887	0.887	0.887	0.887	**0.931**
**Average**		0.751	0.761	0.761	0.762	0.799	0.809	**0.823**

In addition, we further investigated the simulation dataset from the methCancer-gen and benchmark method to assess whether each method approximates the distribution model closely to the original dataset. Utilizing t-distributed stochastic neighbor embedding (t-SNE) [33] method, the original methylome TCGA datasets and the simulation datasets from the methCancer-gen and benchmark were compressed into three-dimensional t-SNE spaces. From the result, the generated dataset from methCancer-gen were clearly separated into individual cancer types, validating that methCancer-gen could capture high-dimensional latent features of original dataset even within the similar cancers showing clusters of partial mixing, while the benchmark method showed sporadic result on those cancers (Supplementary material S4).

## Discussion

Although genome-wide DNA methylation measurement methods such as WGBS has been introduced, still most of the publically available dataset are array-based because of cost-efficiency. Besides, due to the relatively high cost of generating methylome data, the lack of public data issues is still an open problem.

From our modeling and experiments to alleviate the issue, it is proved that methCancer-gen provides more accurate DNA methylation profiles for each cancer type compared to the other method. Five different ML-based classifiers correctly classified the generated dataset from the proposed model to each cancer showing that our model successfully learned latent features and inferred the distribution of each cancer in an unsupervised manner.

methCancer-gen can be used for data augmentation strategy, where utilizing the generated dataset from methCancer-gen as a supplement of real data for model training could indeed improve the performance of a classifier. Up to the certain point, the larger the amount of simulation dataset, the more accurate performance could be achieved. In addition, the generated dataset could be utilized for imputation by replacing missing values (Supplementary material S5).

## Conclusions

In this paper, we presented methCancer-gen, a neural network-based tool for generating DNA methylome samples for user-specified cancer type. The proposed model employs CVAE as a generative model to estimate the distributions that underlie observed methylation values by variational inference while accounting for cancer type. The simulation performance of our model was evaluated with comparison to the benchmark method and the benefit of utilizing methCancer-gen was tested, showing improved performance in both evaluation results. We believe that the methCancer-gen could alleviate the lack of DNA methylation data issue, and promote further epigenetic cancer research.

## Methods

**Fig. 2:**
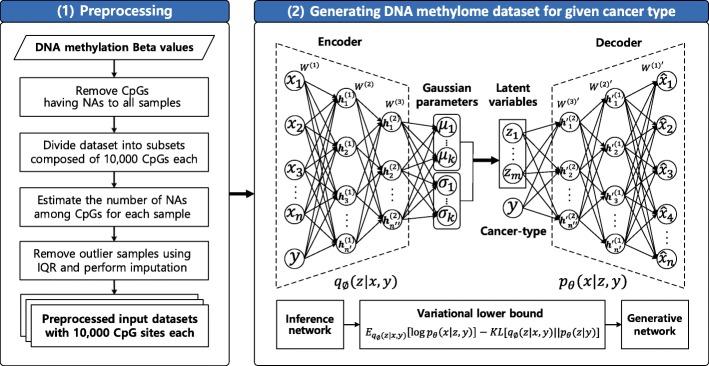
Workflow of the proposed methCancer-gen model based on CVAE using DNA methylation data. It consists of two main phases: (1) Preprocessing to minimize bias caused by high frequency of missing values. (2) Generation of DNA methylation dataset for specified cancer type by CVAE neural network model

With the matrix of DNA methylation beta values and matched cancer type information as input, the methCancer-gen approximates the underlying distribution model of the input data. After model training, methylation beta value for the specified cancer type can be generated as output. Figure 2 depicts a flowchart describing the process.

### Preprocessing

To eliminate the bias caused by a high frequency of missing values during model training, the methCancer-gen provides a four-step preprocessing. First, CpG sites having missing values for all samples were removed. To retrieve maximum data, the dataset is divided into multiple subsets of 10,000 CpGs each. Therefore, samples showing missing values only for specific CpGs within each subset can be utilized for model training. Then, samples having a significant number of missing values are detected as outliers and discarded to minimize bias by applying inter-quartile range (IQR) method [34]. Remaining missing values are imputed with median values.

### Generating DNA methylome dataset for a given cancer type

The methCancer-gen model was constructed based on a CVAE neural network model conditioned on the input observation in VAE, where VAE is a probabilistic generative model combining DNN and variational learning framework. It has been demonstrated that VAE tends to be more stable in model training procedure and producing less obscure output than other generative models, as it is based on clear objective function to optimize based on log-likelihood [35]. Through a process of generating a set of latent variable *z* from the prior distribution *p*_*θ*_(*z*), data *x* is generated from the generative model *p*_*θ*_(*x*|*z*) conditioned on *z* with respect to generative parameter *θ*, where the prior over z is assumed to be the standard normal distribution. To approximate the posterior distribution *p*_*θ*_(*z*|*x*) assumed to be a Gaussian, variational inference is used by introducing a proposal distribution *q*_*ϕ*_(*z*|*x*), known as recognition model, where *ϕ* is the variational parameter. By applying the stochastic gradient variational bayes (SGVB) framework, the Gaussian parameters of VAE, *μ* and *σ* are estimated and the variational lower bound on log-likelihood is used as an objective function :

1 $$ \begin{aligned} & E_{q_{\phi}(z|x)}[\log p_{\theta}(x|z)]-KL[q_{\phi}(z|x)||p_{\theta}(z)] \\ \end{aligned}  $$

, where the first term denotes an expectation over the approximate posterior distribution, called reconstruction error, while the second term is a Kullback-Leibler (KL) divergence term considered as a regularizer. Implemented in a neural network, an encoder referred to as inference network models the recognition model and a decoder defines the conditional probability *p*_*θ*_(*x*|*z*), which is referred to as generative network.

In addition to VAE, CVAE imposes a condition *y* on the *z* and *x*, where the recognition and generation models are extended to *q*_*ϕ*_(*z*|*x*,*y*) and *p*_*θ*_(*x*|*z*,*y*), respectively. In training procedure to maximize the conditional log-likelihood, the parameters of CVAE are estimated, and the variational lower bound on log-likelihood is defined as follows:

2 $$ \begin{aligned} & \log p_{\theta}(x,y) \geq \mathcal{L}_{CVAE} \\ & \quad \quad \quad \quad \ \ \, = \, E_{q_{\phi}(z|x,y)}[\log p_{\theta}(x|z,y)]-KL[q_{\phi}(z|x,y)||p_{\theta}(z|y)] \end{aligned}  $$

After training procedure, through sampling from the learned latent distribution with utilizing the generative network, simulated dataset inferred from input data can be generated. In methCancer-gen, *x* represents the input data of DNA methylation beta values, and *y* is a cancer type.

The methCancer-gen model consists of encoder and decoder with two hidden layers, where the encoder has an architecture of 500 and 250 hidden nodes with fully connected layers and activation functions of empirically-selected exponential linear units (ELUs) [36] and the tanh function [37] were applied. The decoder has a symmetrical structure to encoder extracting 125 latent variables. During the training phase, the model was optimized with the adaptive optimization algorithm, Adam [38] by simultaneously minimizing the reconstruction error and loss. The learning rate and training epoch were set to 1e-3 and 10,000, respectively. methCancer-gen is implemented in python with Tensorflow library (Version 1.8.0) and publicly available at https://github.com/cbi-bioinfo/methCancer-gen.

## Supplementary information

**Additional file 1** Supplementary material S1. (A) Average classification accuracy results for each cancer type based on different classifiers from the simulation performance evaluation in Fig. 1. (B) False positive rate (FPR) of methCancer-gen for each cancer type from the simulation performance evaluation in Fig. 1. To measure the FPR, multi-class datasets are converted to binary classification problems by using one class v.s. others scheme. (C) False negative rate (FNR) of methCancer-gen for each cancer type from the simulation performance evaluation in Fig. 1. To measure the FNR, multi-class datasets are converted to binary classification problems by using one class v.s. others scheme.

**Additional file 2** Supplementary material S2. Average AUC results for each cancer type from the performance evaluation in Fig. 1.To measure the AUC, multi-class datasets are converted to binary classification problems by using one class v.s. others scheme.

**Additional file 3** Supplementary material S3. Average AUC results of the SVM classifier for each cancer type from the second experiment (Table 4) to validate whether training a classifier based on a combined dataset with the original TCGA data and the generate ad data from methCancer-gen could improve the classification performance. Each experiment was repeated five times.

**Additional file 4** Supplementary material S4. t-SNE visualization of the original dataset and simulation dataset from methCancer-gen and the benchmark method is shown.

**Additional file 5** Supplementary material S5. Performance comparison of two SVM classifiers trained by median imputed dataset and imputed dataset using methCancer-gen generated data respectively. 100,000 missing values (NA) for the imputation test were randomly created within 30% samples of TCGAdata.

## Data Availability

TCGA methylation datasets are available from GDC Data Portal (https://portal.gdc.cancer.gov/) and other methylation samples used in the experiment are available from methCNA (http://cgma.scu.edu.cn/MethCNA/). methCancer-gen is publicly available at https://github.com/cbi-bioinfo/methCancer-gen.

## Abbreviations

TCGA: The Cancer Genome Atlas
ENCODE: ENCyclopedia of DNA elements
RRBS: Reduced representation bisulfite sequencing
WGBS: Whole genome bisulfite sequencing
DNN: Deep neural network
VAE: Variational autoencoder
CVAE: Conditional variational autoencoder
IQR: Inter-quartile range
SGVB: Stochastic gradient variational bayes
KL: Kullback-Leibler
ELUs: Empirically-selected exponential linear units
ML: Machine learning
DT: Decision tree
NB: Naive Bayes
RF: Random forest
KNN: K-nearest neighbor
SVM: Support vector machine
AUC: Area under curve
t-SNE: t-distributed stochastic neighbor embedding
