# Supplementary material for: methCancer-gen: a DNA methylome dataset generator for user-specified cancer type based on conditional variational autoencoder
Source: BMC Bioinformatics. 2020 May 11;21:181. doi: 10.1186/s12859-020-3516-8 (PMC7216580; doi:10.1186/s12859-020-3516-8)
Supplement: Supplementary file 1 — Additional file 1 Supplementary material S1. (A) Average classification accuracy results for each cancer type based on different classifiers from the simulation performance evaluation in Fig. 1. (B) False positive rate (FPR) of methCancer-gen for each cancer type from the simulation performance evaluation in Fig. 1. To measure the FPR, multi-class datasets are converted to binary classification problems by using one class v.s. others scheme. (C) False negative rate (FNR) of methCancer-gen for each cancer type from the simulation performance evaluation in Fig. 1. To measure the FNR, multi-class datasets are converted to binary classification problems by using one class v.s. others scheme. [file 12859_2020_3516_MOESM1_ESM.pdf]

Supplementary material S1.

(A) Average classification accuracy results for each cancer type based on different classifiers from the simulation performance evaluation in Figure 1.

[methCancer-gen]

	DT	NB	KNN	RF	SVM
**BLCA**	0.902	1.000	0.991	0.983	1.000
**BRCA**	0.202	1.000	1.000	0.993	1.000
**CESC**	0.040	0.000	0.970	0.665	1.000
**COAD**	0.770	1.000	1.000	0.978	1.000
**ESCA**	0.182	0.960	0.050	0.116	1.000
**GBM**	0.569	0.980	0.895	0.798	1.000
**HNSC**	0.821	1.000	1.000	0.996	1.000
**KIRC**	0.938	1.000	1.000	1.000	1.000
**KIRP**	0.853	0.999	1.000	0.975	1.000
**LGG**	0.932	1.000	1.000	0.998	1.000
**LIHC**	0.995	1.000	1.000	1.000	1.000
**LUAD**	0.976	1.000	1.000	1.000	1.000
**LUSC**	0.671	0.979	1.000	0.814	1.000
**MESO**	0.785	0.663	0.788	0.893	1.000
**PAAD**	0.844	0.959	1.000	0.930	1.000
**PCPG**	0.973	0.990	1.000	1.000	1.000
**PRAD**	0.940	1.000	1.000	1.000	1.000
**READ**	0.334	0.083	0.000	0.246	0.332
**SARC**	0.807	1.000	0.264	0.988	1.000
**SKCM**	0.873	0.999	0.985	0.963	1.000
**STAD**	0.713	0.999	1.000	0.969	1.000
**TGCT**	0.429	0.999	1.000	0.688	1.000
**THCA**	0.969	1.000	1.000	1.000	1.000
**THYM**	0.658	0.709	1.000	0.846	1.000
**UCEC**	0.133	0.000	1.000	0.912	1.000

[Benchmark]

	DT	NB	KNN	RF	SVM
**BLCA**	0.630	1.000	1.000	0.943	1.000
**BRCA**	0.166	1.000	1.000	0.907	1.000
**CESC**	0.051	0.000	0.999	0.602	1.000
**COAD**	0.581	1.000	1.000	0.771	1.000
**ESCA**	0.224	0.319	0.197	0.209	1.000
**GBM**	0.467	0.626	0.900	0.689	1.000
**HNSC**	0.617	1.000	1.000	0.833	1.000
**KIRC**	0.780	1.000	1.000	0.980	1.000
**KIRP**	0.651	1.000	1.000	0.879	1.000
**LGG**	0.856	1.000	1.000	0.992	1.000
**LIHC**	0.944	1.000	1.000	1.000	1.000
**LUAD**	0.865	1.000	1.000	1.000	1.000
**LUSC**	0.529	0.913	1.000	0.590	1.000
**MESO**	0.647	0.139	0.800	0.765	1.000
**PAAD**	0.683	0.640	1.000	0.773	1.000
**PCPG**	0.893	1.000	1.000	0.967	1.000
**PRAD**	0.977	1.000	1.000	1.000	1.000
**READ**	0.387	0.000	0.000	0.384	0.312
**SARC**	0.640	1.000	0.182	0.780	1.000
**SKCM**	0.718	0.906	1.000	0.864	1.000
**STAD**	0.471	1.000	1.000	0.699	1.000
**TGCT**	0.405	1.000	1.000	0.615	1.000
**THCA**	0.962	1.000	1.000	0.999	1.000
**THYM**	0.624	0.786	1.000	0.819	1.000
**UCEC**	0.119	0.000	1.000	0.856	1.000

(B) False positive rate (FPR) of methCancer-gen for each cancer type from the simulation performance evaluation in Figure 1. To measure the FPR, multi-class datasets are converted to binary classification problems by using one class v.s. others scheme.

	**DT**	**NB**	**KNN**	**RF**	**SVM**
**BLCA**	0.027	0.026	0.001	0.015	0.000
**BRCA**	0.027	0.009	0.000	0.008	0.000
**CESC**	0.005	0.000	0.000	0.001	0.000
**COAD**	0.026	0.038	0.042	0.030	0.030
**ESCA**	0.023	0.005	0.000	0.004	0.000
**GBM**	0.008	0.000	0.000	0.000	0.000
**HNSC**	0.040	0.036	0.034	0.032	0.000
**KIRC**	0.011	0.000	0.000	0.003	0.000
**KIRP**	0.006	0.000	0.000	0.001	0.000
**LGG**	0.017	0.001	0.004	0.009	0.000
**LIHC**	0.003	0.000	0.000	0.001	0.000
**LUAD**	0.003	0.000	0.012	0.000	0.000
**LUSC**	0.037	0.025	0.003	0.010	0.000
**MESO**	0.009	0.000	0.000	0.001	0.000
**PAAD**	0.017	0.000	0.026	0.001	0.000
**PCPG**	0.004	0.000	0.000	0.001	0.000
**PRAD**	0.003	0.000	0.000	0.001	0.000
**READ**	0.005	0.000	0.000	0.000	0.000
**SARC**	0.011	0.007	0.000	0.003	0.000
**SKCM**	0.003	0.000	0.000	0.000	0.000
**STAD**	0.019	0.007	0.005	0.014	0.000
**TGCT**	0.003	0.000	0.000	0.000	0.000
**THCA**	0.005	0.000	0.000	0.000	0.000
**THYM**	0.002	0.000	0.000	0.000	0.000
**UCEC**	0.009	0.000	0.000	0.001	0.000
**Average**	0.013	0.006	0.005	0.005	0.001

(C) False negative rate (FNR) of methCancer-gen for each cancer type from the simulation performance evaluation in Figure 1. To measure the FNR, multi-class datasets are converted to binary classification problems by using one class v.s. others scheme.

	DT	NB	KNN	RF	SVM
**BLCA**	0.098	0.000	0.009	0.017	0.000
**BRCA**	0.798	0.000	0.000	0.007	0.000
**CESC**	0.960	1.000	0.030	0.335	0.000
**COAD**	0.230	0.000	0.000	0.022	0.000
**ESCA**	0.818	0.040	0.950	0.884	0.000
**GBM**	0.431	0.020	0.105	0.202	0.000
**HNSC**	0.179	0.000	0.000	0.004	0.000
**KIRC**	0.062	0.000	0.000	0.000	0.000
**KIRP**	0.147	0.001	0.000	0.025	0.000
**LGG**	0.068	0.000	0.000	0.002	0.000
**LIHC**	0.005	0.000	0.000	0.000	0.000
**LUAD**	0.024	0.000	0.000	0.000	0.000
**LUSC**	0.329	0.021	0.000	0.186	0.000
**MESO**	0.215	0.337	0.212	0.107	0.000
**PAAD**	0.156	0.041	0.000	0.070	0.000
**PCPG**	0.027	0.010	0.000	0.000	0.000
**PRAD**	0.060	0.000	0.000	0.000	0.000
**READ**	0.666	0.917	1.000	0.754	0.730
**SARC**	0.193	0.000	0.736	0.012	0.000
**SKCM**	0.127	0.001	0.015	0.037	0.000
**STAD**	0.287	0.001	0.000	0.031	0.000
**TGCT**	0.571	0.001	0.000	0.312	0.000
**THCA**	0.031	0.000	0.000	0.000	0.000
**THYM**	0.342	0.291	0.000	0.154	0.000
**UCEC**	0.867	1.000	0.000	0.088	0.000
**Average**	0.308	0.147	0.122	0.130	0.029
