# Supplementary material for: methCancer-gen: a DNA methylome dataset generator for user-specified cancer type based on conditional variational autoencoder
Source: BMC Bioinformatics. 2020 May 11;21:181. doi: 10.1186/s12859-020-3516-8 (PMC7216580; doi:10.1186/s12859-020-3516-8)
Supplement: Supplementary file 2 — Additional file 2 Supplementary material S2. Average AUC results for each cancer type from the performance evaluation in Fig. 1.To measure the AUC, multi-class datasets are converted to binary classification problems by using one class v.s. others scheme. [file 12859_2020_3516_MOESM2_ESM.pdf]

Supplementary material S2.

Average AUC results for each cancer type from the performance evaluation in Figure 1.
To measure the AUC, multi-class datasets are converted to binary classification problems by using one class v.s. others scheme.

[methCancer-gen]

	DT	NB	KNN	RF	SVM
**BLCA**	0.937	0.987	1.000	0.984	1.000
**BRCA**	0.588	0.996	1.000	0.992	1.000
**CESC**	0.518	0.500	0.985	0.832	1.000
**COAD**	0.872	0.981	0.979	0.974	0.986
**ESCA**	0.579	0.977	0.599	0.556	1.000
**GBM**	0.780	0.990	0.948	0.899	1.000
**HNSC**	0.890	0.982	0.983	0.982	1.000
**KIRC**	0.964	1.000	1.000	0.999	1.000
**KIRP**	0.924	1.000	1.000	0.987	1.000
**LGG**	0.958	1.000	0.999	0.995	1.000
**LIHC**	0.996	1.000	1.000	1.000	1.000
**LUAD**	0.987	1.000	0.994	1.000	1.000
**LUSC**	0.817	0.977	0.999	0.902	1.000
**MESO**	0.888	0.832	0.894	0.946	1.000
**PAAD**	0.914	0.980	0.987	0.964	1.000
**PCPG**	0.984	0.995	1.000	1.000	1.000
**PRAD**	0.968	1.000	1.000	1.000	1.000
**READ**	0.665	0.542	0.500	0.623	0.656
**SARC**	0.898	0.997	0.632	0.993	1.000
**SKCM**	0.935	1.000	0.993	0.981	1.000
**STAD**	0.847	0.996	0.997	0.977	1.000
**TGCT**	0.713	1.000	1.000	0.844	1.000
**THCA**	0.982	1.000	1.000	1.000	1.000
**THYM**	0.828	0.855	1.000	0.923	1.000
**UCEC**	0.562	0.500	1.000	0.956	1.000
**Average**	0.840	0.923	0.940	0.932	0.986

[Benchmark]

	DT	NB	KNN	RF	SVM
**BLCA**	0.798	0.992	1.000	0.955	1.000
**BRCA**	0.566	0.991	1.000	0.946	1.000
**CESC**	0.520	0.500	1.000	0.799	1.000
**COAD**	0.776	0.979	0.979	0.871	0.985
**ESCA**	0.595	0.657	0.599	0.597	1.000
**GBM**	0.726	0.813	0.950	0.844	1.000
**HNSC**	0.793	0.998	0.994	0.905	1.000
**KIRC**	0.880	1.000	1.000	0.986	1.000
**KIRP**	0.820	1.000	1.000	0.938	1.000
**LGG**	0.920	0.992	0.998	0.989	1.000
**LIHC**	0.968	1.000	1.000	0.999	1.000
**LUAD**	0.929	0.996	0.989	1.000	1.000
**LUSC**	0.744	0.952	0.998	0.787	1.000
**MESO**	0.819	0.570	0.900	0.881	1.000
**PAAD**	0.832	0.820	0.993	0.885	1.000
**PCPG**	0.943	1.000	1.000	0.982	1.000
**PRAD**	0.985	1.000	1.000	0.999	1.000
**READ**	0.688	0.500	0.500	0.688	0.635
**SARC**	0.813	0.985	0.591	0.886	1.000
**SKCM**	0.856	0.953	1.000	0.932	1.000
**STAD**	0.722	0.956	0.987	0.842	1.000
**TGCT**	0.699	1.000	1.000	0.807	1.000
**THCA**	0.977	1.000	1.000	0.999	1.000
**THYM**	0.809	0.893	1.000	0.909	1.000
**UCEC**	0.556	0.500	1.000	0.927	1.000
**Average**	0.789	0.882	0.939	0.894	0.985
