# Supplementary material for: methCancer-gen: a DNA methylome dataset generator for user-specified cancer type based on conditional variational autoencoder
Source: BMC Bioinformatics. 2020 May 11;21:181. doi: 10.1186/s12859-020-3516-8 (PMC7216580; doi:10.1186/s12859-020-3516-8)
Supplement: Supplementary file 3 — Additional file 3 Supplementary material S3. Average AUC results of the SVM classifier for each cancer type from the second experiment (Table 4) to validate whether training a classifier based on a combined dataset with the original TCGA data and the generate ad data from methCancer-gen could improve the classification performance. Each experiment was repeated five times. [file 12859_2020_3516_MOESM3_ESM.pdf]

Supplementary material S3.

Average AUC results of the SVM classifier for each cancer type from the second experiment (Table 4) to validate whether training a classifier based on a combined dataset with the original TCGA data and the generatead data from methCancer-gen could improve the classification performance. Each experiment was repeated five times.

Cancer	TCGA only	TCGA & benchmark			TCGA & methCancer-gen		
		Number of generated dataset for each cancer type					
		**100**	**200**	**300**	**100**	**200**	**300**
**BLCA**	0.891	0.894	0.894	0.894	0.895	0.897	0.903
**COAD**	0.959	0.959	0.959	0.959	0.964	0.974	0.974
**GBM**	0.986	0.986	0.986	0.986	0.986	0.986	0.986
**KIRC**	0.866	0.867	0.867	0.867	0.867	0.867	0.867
**LUAD**	0.854	0.873	0.873	0.873	0.896	0.900	0.902
**PAAD**	0.560	0.575	0.575	0.578	0.690	0.708	0.712
**PRAD**	0.850	0.850	0.850	0.850	0.850	0.850	1.000
**SKCM**	0.943	0.943	0.943	0.943	0.943	0.943	0.965
**Average**	0.864	0.868	0.868	0.869	0.886	0.891	0.914
