# Supplementary material for: methCancer-gen: a DNA methylome dataset generator for user-specified cancer type based on conditional variational autoencoder
Source: BMC Bioinformatics. 2020 May 11;21:181. doi: 10.1186/s12859-020-3516-8 (PMC7216580; doi:10.1186/s12859-020-3516-8)

Supplementary material S4.

t-SNE visualization of the original dataset and simulation dataset from methCancer-gen and the benchmark method is shown.

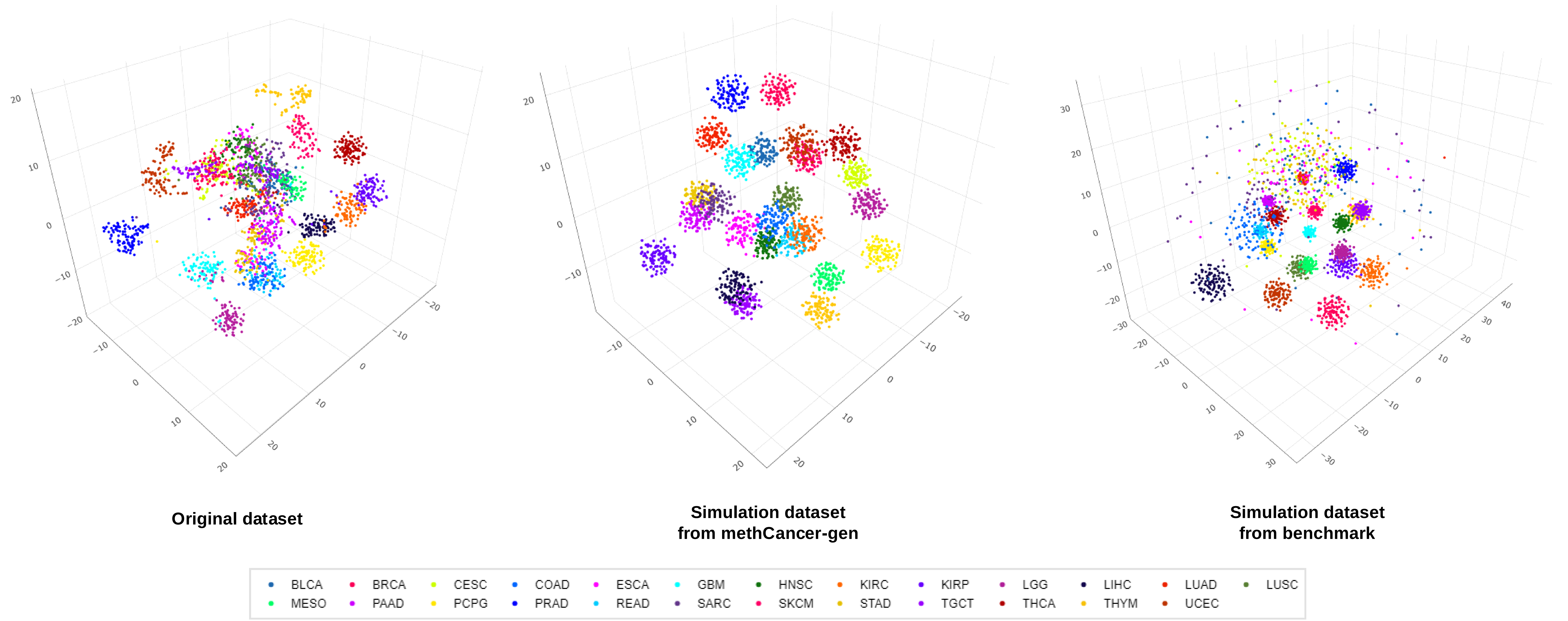

Supplement: Supplementary file 4 — Additional file 4 Supplementary material S4. t-SNE visualization of the original dataset and simulation dataset from methCancer-gen and the benchmark method is shown. [file 12859_2020_3516_MOESM4_ESM.pdf]
