# Supplementary material for: methCancer-gen: a DNA methylome dataset generator for user-specified cancer type based on conditional variational autoencoder
Source: BMC Bioinformatics. 2020 May 11;21:181. doi: 10.1186/s12859-020-3516-8 (PMC7216580; doi:10.1186/s12859-020-3516-8)
Supplement: Supplementary file 5 — Additional file 5 Supplementary material S5. Performance comparison of two SVM classifiers trained by median imputed dataset and imputed dataset using methCancer-gen generated data respectively. 100,000 missing values (NA) for the imputation test were randomly created within 30% samples of TCGAdata. [file 12859_2020_3516_MOESM5_ESM.pdf]

Supplementary material S5.

Performance comparison of two SVM classifiers trained by median imputed dataset and imputed dataset using methCancer-gen generated data respectively. 100,000 missing values (NA) for the imputation test were randomly created within 30% samples of TCGA data.

Accuracy of SVM	median imputation	methCancer-gen
Test 1	0.781	0.805
Test 2	0.780	0.803
Test 3	0.781	0.807
Test 4	0.779	0.805
Test 5	0.780	0.803
**Average**	0.781	**0.805**
